# That's a BIG deal: Identification of a regulator in hypoxia responses and suberin deposition

**DOI:** 10.1093/plcell/koae138

**Published:** 2024-05-01

**Authors:** Margot Raffeiner

**Affiliations:** Assistant Features Editor, The Plant Cell, American Society of Plant Biologists; Faculty of Biology and Biotechnology, Ruhr University Bochum, Bochum 44801, Germany

If you believe the countless advertisements that constantly bombard you, the first step to a happy life is leading a healthy life. The basis for a healthy life of any organism, including plants, is found at the cellular level, where mechanisms of protein synthesis and degradation act in concert to guarantee protein homeostasis, or proteostasis for short. Many misfolded, short-lived, or nonfunctional proteins are degraded via the ubiquitin-26S proteasome system (UPS) that plays a major role in the maintenance of proteostasis. Modifications to the amino-terminal (Nt) residue of numerous proteins are known to determine protein stability; N-degrons are specific Nt modifications that tag proteins for degradation (via the UPS or other pathways), created by the cleavage activity of endopeptidases or by modifications introduced by other enzyme classes (e.g. arginylation of the Nt residue by arginyl tRNA transferases). N-degron pathways form an important entry point to the UPS (Holdsworth et al. 2020). Some plant E3 ligases, also known as N-recognins, specifically recognize different N-degrons and catalyze their ubiquitylation and subsequent degradation via the UPS. PROTEOLYSIS6 (PRT6) shows high specificity toward basic residues (e.g. Arg), while PRT1 has been shown to preferentially recognize aromatic residues (e.g. Phe).

In recent years it has become more evident that N-degron–mediated protein degradation has broad implications in the regulation of plant developmental processes as well as biotic and abiotic stress responses (Holdsworth et al. 2020). In 2011 Gibbs and colleagues, alongside Licausi and colleagues, found that the PRT6/N-degron pathway has a role in regulating plant response to hypoxia (specifically low oxygen stress) by identifying hypoxia-associated Ethylene Response Factor (ERF) Group VII transcription factors (ERFVII TFs) as PRT6 substrates (Gibbs et al. 2011; Licausi et al. 2011). Under normoxia (nonstress normal oxygen levels), these ERFVII TFs are oxidized, arginylated, and therefore recognized by PRT6 and subsequently degraded via the 26S proteasome. However, they are stabilized when oxygen availability is scarce, allowing the expression of hypoxia-responsive genes.

In this issue, **Hongtao Zhang and colleagues** (Zhang et al. 2024) present another player in the hypoxia response, BIG/DARK OVEREXPRESSION OF CAB1/TRANSPORT INHIBITOR RESPONSE 3 (BIG/DOC1/TIR3; BIG hereafter). BIG is proposed to act as a plant N-recognin working in concert with PRT6. BIG owes its name to its extraordinary protein size of 0.5 MDa (Gil et al. 2001), but its numerous biological functions are similarly impressive (Zhang et al. 2024). To investigate a role for BIG in Arg/N-degron pathways, Zhang et al. generated Arabidopsis double mutant lines lacking both a functional BIG protein and 1 of the 2 functional N-degron pathway E3 ligases, PRT6 or PRT1.

In *prt6 big* mutants, the stability of model substrates (Figure), as well as different physiological PRT6 substrates, was enhanced compared to Arabidopsis wild-type Columbia-0 (Col-0) or single mutant plants. In turn, this indicated that BIG supports PRT6-mediated target degradation. Two of the physiological substrates used in this study were representative ERFVII TFs involved in hypoxia gene regulation. In line with elevated protein accumulation of these TFs in the double mutants, the authors observed a significantly enhanced induction of core hypoxia gene expression and respective protein abundance. A more detailed investigation revealed that BIG influences the hypoxia response exclusively through RELATED TO APETALA (RAP)-type ERFVII TFs and that arginylation allows BIG to exert its N-recognin function on these target substrates.

**Figure.**
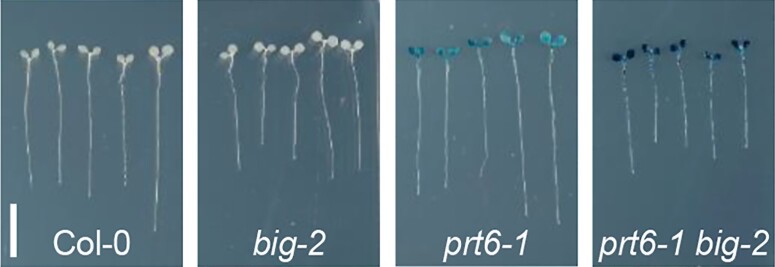
BIG influences the stability of Arg/N-degron pathway substrates. An *Escherichia coli* GUS reporter protein that reveals an Arginine (R) at its N terminus upon cleavage by a specific protease was used as a model substrate (R-GUS reporter). GUS reporter activity was assessed by histochemical staining and revealed enhanced stability of the R-GUS substrate in Arabidopsis *prt6-1 big-2* double mutants compared to the *prt6-1* single mutant. No stabilization of the model substrate was observed in Col-0 control plants and the *big-2* single mutant. Adapted from Zhang et al. (2024), Figure 1G.

Large-scale transcriptome analysis of root material from Col-0 and different mutants revealed extensive remodeling of the mutant's transcriptome. It not only confirmed that hypoxia-related genes were highly enriched in the *prt6* single and *prt6 big* double mutants but also identified many components involved in suberin biosynthesis and transport that were significantly downregulated in gene expression in those mutants. This indicates that the BIG/PRT6-mediated N-degron pathway has an additional role in controlling the suberin deposition process in Arabidopsis.

This study not only unraveled previously unknown regulatory functions of BIG in different biological contexts but also proposes BIG as a newly identified plant N-recognin, shedding light on its putative biochemical activity.

## Dive Curated Terms

The following phenotypic, genotypic, and functional terms are of significance to the work described in this paper:

PRT1 Gramene: At3g24800
PRT1 Araport: At3g24800

PRT6 Gramene: At5g02310
PRT6 Araport: At5g02310
